# Nutritional Quality of the European Spiny Lobster *Palinurus elephas* (J.C. Fabricius, 1787) (Achelata, Palinuridae) and the Non-Indigenous Northern Brown Shrimp *Penaeus aztecus* Ives, 1891 (Dendrobranchiata, Penaeidae)

**DOI:** 10.3390/foods10102480

**Published:** 2021-10-17

**Authors:** Thodoros E. Kampouris, Adamantia Asimaki, Dimitris Klaoudatos, Athanasios Exadactylos, Ioannis T. Karapanagiotidis, Ioannis E. Batjakas

**Affiliations:** 1 Department of Marine Sciences, School of the Environment, University of the Aegean, University Hill, Lesvos Island, 811 00 Mytilene, Greece; jbatzakas@marine.aegean.gr; 2 Department of Ichthyology and Aquatic Environment, School of Agricultural Sciences, University of Thessaly, Fytokou Street, 384 45 Volos, Greece; mantwasim@gmail.com (A.A.); dklaoud@uth.gr (D.K.); exadact@uth.gr (A.E.); ikarapan@uth.gr (I.T.K.)

**Keywords:** spiny lobster, northern brown shrimp, proximate composition, fatty acids, threatened species, non-indigenous species, Aegean Sea, Greece

## Abstract

The European spiny lobster is a species of great commercial value, yet a limited scientific knowledge exists on its biology, ecology, and physiology, especially for the stocks from east Mediterranean waters. The northern brown shrimp, a non-indigenous established species, is commercially exploited in regions of the Mediterranean Sea. Both species’ proximate composition and fatty acid profile were assessed for the first time in the Mediterranean region, exhibiting an overall significant statistical difference. Protein, fat, and energy contents were significantly higher in the northern brown shrimp, whereas moisture and ash contents were significantly higher in the European spiny lobster. The proximate composition for both species was well within the reported range for other lobster and prawn species in the Mediterranean Sea.

## 1. Introduction

Shellfish are food sources of high nutritional value, offering high amounts of proteins, minerals, vitamins, n-3 fatty acids, and amino acids e.g., [1,2,3,4,5]. It is generally accepted that shellfish consumption positively contributes to the prevention of several diseases, such as cardiovascular, inflammatory, heart diseases, and cancer [2,3,4,6]. Studies regarding the proximate, chemical, or total composition typically focus on commercial fishery resources [3,7]. Furthermore, studies also focus on well-established cultured species and on the improvement of farming methodologies and techniques e.g., [8,9] and species of future aquaculture potential [10,11,12,13,14].

Proximate composition can be affected by developmental stage, as indicated by lipid content decreasing during the development of *Homarus gammarus* [15]. Sex also plays an important role, with different sexes of *H. gammarus* and *H. americanus* exhibiting significant differences in their proximate composition profiles [3]. Furthermore, Castille and Lawrence [16] indicated that reproduction can affect lipid and protein content as indicated by lipid and protein content increase in the ovaries and decrease in muscle tissue during gonad maturation for *Penaeus aztecus* and *P. setiferus*. Moreover, the nutritional value of some prawn species such as *P. kerathurus* can be affected by fish farms, since it has been demonstrated that they can feed with fish feeds losses [17].

The European spiny lobster, *Palinurus elephas* (J.C. Fabricius, 1787), is a highly esteemed and sought-after commercial species, under high fishing pressure, harvested in the Mediterranean and adjacent Atlantic waters from north Africa to Scotland [18]. Despite its high commercial value, available fisheries data are regional and limited, especially for the stocks from east Mediterranean waters [19,20]. Recent research focused on the species’ acoustic behavior [21,22,23] and activity patterns [24]; however, important aspects of its biology, physiology, and ecology are yet unknown [18], and even though the species is of great economic importance, its nutritional quality has not been yet assessed.

The northern brown shrimp *Penaeus aztecus* Ives, 1891, is an important commercial penaeid shrimp in the US [25] native to American Atlantic coast from Massachusetts USA to Yucatan Peninsula Mexico [26]. The first Mediterranean sighting was recorded in 2009 off the coast of Antalya, Turkey [27]. Since then, the species quickly expanded its distribution in many sub-basins of the Mediterranean Sea including Levantine, Aegean, Ionian, Adriatic, Tyrrhenian seas, and the Gulf of Lion. The study of Abdulrraziq et al. [28] provides the most updated data on the species’ current distribution in Mediterranean waters. Moreover, *P. aztecus* was reported from the Black Sea [29] and from Scheldt estuary, Belgium [30]. The species is commercially exploited as a fishery resource in parts of the Mediterranean Sea, including Greece [31,32] and Sicily, Italy [33]. However, available data are limited to length–weight relationships [34,35].

The introduction vector of *P. aztecus* has been debated, and both shipping (though ballast water) and accidental aquaculture escapes were suggested. Given the fact that prawn culture in the Mediterranean region is quite limited, and in regard to other species such as *P. japonicus* and *P. semisulcatus* in Turkey and *P. vannamei* in Egypt and not *P. aztecus* [36], and references within, the probability of an aquaculture escape seems to be quite thin. Furthermore, *P. aztecus* was caught for the first time at Thermaikos Gulf around 2006–2007, and since then, individuals are sold to the market mixed with native pawn species, depending on their size. Larger *P. aztecus* are sold mixed with *P. kerathurus* and smaller ones are indeed mixed with *Parapenaeus longirostris* (H. Lucas, 1846) (Kampouris, unpublished data). 

The aim of the present study was to assess and compare for the first time the proximate composition and fatty acid profiles of *P. elephas* and *P. aztecus*. Furthermore, we compared the proximate composition of each studied species with published data from decapod species present in the Mediterranean. 

## 2. Materials and Methods

### 2.1. Captured Samples

Spiny lobsters were caught within the National Marine Park of Alonissos & Northern Sporades Islands (northwest Aegean Sea, Greece) and particularly in the coastal waters of Psathoura, Gioura, and Kyra Panagia islands. The isles of Psathoura (Zone A2), Gioura (Zone A3), and Kyra Panagia (Zone A4) are within the area of the National Marine Park of Alonissos Northern Sporades that professional coastal fishing is permitted [36]. Trammel and tangle nets 3–5 km in length were set over rocky substrate using a 9.5 m long fishing vessel at a depth ranging between 40 and 100 m. Soak time varied between 10 and 12 h, depending on weather conditions. The net mesh size used was 100 mm (knot to knot). 

Prawn individuals were caught from the west shores of Thermaikos Gulf, northwest Aegean Sea, Greece. The specimens were provided by a professional artisanal fisherman using a 10 m long vessel. Tangle nets, specially modified for shrimps and prawns, nets of 2–3 km in length, over sandy and muddy substrates, were used. The mesh size was 20 mm (knot to knot). Soaking time was from 8 to 10 h, depending on weather conditions.

### 2.2. Proximate Composition

Proximate composition was assessed to determine the nutrient composition of the carcass (tail muscle) of spiny lobsters and prawns based on methods described in AOAC [37]. In total, ten specimens of each species were used. Thermal drying to constant weight in an oven at 105 °C for 24 h was applied to determine moisture content. Crude protein content was determined by Kjeldahl analyses (N × 6.25; Βehr Labor-Technik, Düsseldorf, Germany). Crude fat was determined by exhaustive Soxhlet extraction using petroleum ether (40–60 °C, BP) using a Soxtherm Multistat/SX PC (Sox-416 Macro, Gerhard, Germany). Ash content was determined by dry ashing in porcelain crucibles in a muffle furnace (Nabertherm L9/12/C6, Lilienthal, Germany) at 600 °C for 5 h, and gross energy content was determined adiabatically using an IKA oxygen bomb calorimeter (C5000, IKA Werke, Staufen, Germany).

### 2.3. Fatty Acids

The fatty acid profile of the total lipid from the muscle tails of spiny lobsters and prawns was determined from six specimens of each species. Fatty acid methyl esters (FAME) were prepared by acid catalyzed transesterification, as described by Bouras et al. [38]. FAME was purified by thin layer chromatography (TLC) on 20 × 20 glass plates pre-coated with silica gel G and then separated by gas–liquid chromatography using a Perkin Elmer Clarus 680 coupled with a Col-Elite FameWax capillary column (30 mm × 0.25 mm id, film thickness 0.25 μm (Perkin Elmer, Waltham, MA, USA)) equipped with a flame ionization detector (FID). Hydrogen was used as carrier gas; the injector temperature was set at 240 °C with a split ratio of 1:10 at a total flow rate of 5 mL/min. The temperature was programmed from 60 to 190 °C at a rate of 20 °C/min and maintained for 5 min and from 190 to 240 °C at a rate of 5 °C/min and maintained for 10 min. Identification of individual FAME was conducted by comparison to known standards (FAME MIX 37, Sigma-Aldrich, St. Louis, MO, USA). Peak areas were quantified with reference to the peak area of 17:0 internal standard and chromatograms were analyzed using TotalChrom software (v. 6.3, Perkin Elmer).

### 2.4. Statistical Analysis

The null hypothesis of no significant differences in the proximate composition and fatty acid profiles between species was assessed with Student’s t-test. Normal distribution was assessed using the Shapiro–Wilk normality test. Bartlett’s and Levene’s tests were used to assess homogeneity of variance. When the assumption of normality was not met, the non-parametric Mann–Whitney U test was used. When the assumption of homoscedasticity was not met, the parametric Welch’s unequal variances t-test was used. Comparison of estimated proximate composition between each studied species and estimated values from decapod crustaceans present in the Mediterranean was performed with one sample *t*-test. Statistical analyses were performed using jamovi [39,40] at an alpha level of 0.05.

## 3. Results

The proximate composition and statistical comparison of the tail carcass of *Palinurus elephas* and *Penaeus aztecus* is presented in Table 1.

Proximate composition between studied species overall exhibited a significant differential pattern. Significantly higher crude protein, crude fat, and gross energy contents were recorded in the northern brown shrimp, whereas moisture and ash content were significantly higher in the European spiny lobster.

The proximate composition of established decapod crustaceans in the Mediterranean and their comparison with estimated values for each studied species is illustrated in Table 2 and Table 3.

The fatty acid profiles of *P. elephas* and *P. aztecus* are shown in Table 4. A total number of twenty-five fatty acids of various chain lengths and saturation levels were identified by gas–liquid chromatography. Of the saturated fatty acids (SFA), palmitic acid (16:0) and stearic acid (18:0) were found to be dominant in both species. These fatty acids occur naturally in crustaceans and fish lipids, being the major products of the fatty acid synthetase system [45]. The 16:0 and 18:0, as well as 18:1n-9, are also the major substrates for b-oxidation and production of metabolic energy in shellfish and fish [45]. All other SFA were minor components, although C14:0 and C15:0 comprised 1.2–1.6% (Table 4). Oleic acid (18:1n-9) was the major monounsaturated fatty acid (MUFA) in both species followed by C16:1n-7 and 18:1n-7. These fatty acids naturally occur in large amounts in the aquatic food webs, and they can also be synthesized de novo by fish and shellfish [45]. Gadoleic acid (20:1n-9), cetoleic acid (22:1n-11), and nervonic acid (24:1n-9) are commonly found in small amounts in all shellfish oils, as also observed in the present study. As far as the polyunsaturated fatty acids (PUFA) are concerned, arachidonic acid (20:4n-6), eicosapentaenoic acid (20:5n-3), and docosahexaenoic acid (22:6n-3) were the most abundant PUFA in the muscle lipids of both species, which is followed by linoleic acid (18:2n-6). The importance of these fatty acids, especially of n-3 highly unsaturated fatty acids, in fish and crustacean nutrition is well known [46,47], while they are critical nutrients for human nutrition [48]. In crustaceans, it has been shown that 20:5n-3 and 22:6n-3 play significant roles in the reproduction process [49], as well as in molting and growth [50]. 

In general, both species were characterized by higher fractions of SFA and monounsaturated (MUFA) than of polyunsaturated fatty acids (PUFA). In comparison, both species had similar contents of total saturated and monounsaturated fatty acids, and of each individual saturated and monounsaturated fatty acid, except that of 16:1n-7 where *P. elephas* exhibited a significantly higher value. *P. aztecus* had a much lower content of total n-6 PUFA and a much higher content of n-3 PUFA compared to *P. elephas* that exhibited very poor levels of the latter fatty acid group. In particular, the tail lipids of *P. elephas* were characterized by higher contents of arachidonic acid (20:4n-6), while those of *P. aztecus* were characterized by higher contents of eicosapentaenoic acid (EPA, 20:5n-3) and docosahexaenoic acid (DHA, 22:6n-3). Thus, the n-3/n-6 PUFA ratio was much higher in the latter species.

## 4. Discussion

Although European spiny lobster is an esteemed species [51] of high commercial value [19,52], its proximate composition had not been assessed so far. Similarly, the northern brown shrimp is an alien species in the Mediterranean with proximate composition not assessed after its introduction in 2009 [27]. Results of the present study indicated significantly higher protein, fat, and energy contents in the northern brown shrimp, whereas moisture and ash contents were significantly higher in the European spiny lobster. The proximate compositions of both species were well within the range reported for other lobsters and shrimps in the Mediterranean Sea [1,2,3,4,42,43] and within those of *P. aztecus* from the USA [41]. Furthermore, the proximate composition of both studied species exhibited similarities with the stomatopod *Squilla mantis* (Linnaeus, 1758) [53], the white seabream *Diplodus sargus* (Linnaeus, 1758), and the brown meagre *Sciaena umbra* Linnaeus, 1758, with respect to lipid and protein contents [13].

The proximate composition, in lobsters, is affected by the feeding preferences. For instance, carnivorous spiny lobsters demand higher protein and lipid contents [49]. The European spiny lobster is considered as an omnivorous species that feeds mainly on mollusks, crustaceans, and sea urchins [54], which could explain the significantly lower fat, protein, and energy contents of the present spiny lobster specimens. In addition, seasons may be an important factor that could have an impact at the proximate composition of lobster species e.g., [1]. However, all spiny lobster specimens of the present study were collected during the main fishing season (June–August 2019). It has been proven that the growth stage could be an important factor that affects the proximate composition of lobster species such as *H. gammarus*, but all the specimens of the present study were adults. 

The European spiny lobster is a species with limited available published data, and further research should aim to address issues such as potential differentiations in season, locality, sex, and/or adult or juvenile individuals that may depict fluctuations on the species’ proximate or total composition. However, exempting locality and sex, the other variable factors will not be easy to be thoroughly assessed, since the regulations regarding the lobster fishery in Greece set strict restrictions on the minimum landing sizes, the landing of ovigerous females, and the fishing prohibition period [19]. Perhaps all the above obstacles could be overcome with appropriate experimental sampling permits from the national authorities and funding in order to cover survey expenses.

*Palinurus elephas* was formerly commonly abundant near the coast but is now rare at depths less than 40 m, while its past productive fishery virtually depleted [18]. It was believed that viable fisheries remain intact only in the most remote fishing grounds of the Mediterranean Sea. Based on recent findings, it seems that in the Aegean Sea, some populations remain in proximity to the coastal areas of Greece’s mainland (Chalkidiki Peninsula, northeast Aegean coasts) [19]. In addition, Kampouris et al. [19] demonstrated that systematic stock monitoring is urgently required to assess the exploitation rates of a threatened fishery resource such as the European spiny lobster. The high unit value together with its biological and ecological characteristics in the Eastern Atlantic Ocean and Mediterranean Sea makes it a highly vulnerable species to overexploitation. Its low growth rate, long lifespan, and low fecundity compared to most of the other commercial spiny lobsters [18] coupled with the small amplitude of adult movements further contribute to the overexploitation of the species, and it further underlies the need to define such complicated genetic stock composition patterns [55,56,57].

The promotion of an alien species for human consumption, when possible, is a standard strategy of non-indigenous species management. For instance, in the Mexican Caribbean region, recent studies indicated that the invasive lionfish, *Pterois volitans* (Linnaeus, 1758), could become a valuable food source [58]. Studies from Iran demonstrated the potential of the invasive shrimp species *Macrobrachium nipponense* (De Haan, 1849) as a fishery resource [59]. In the Mediterranean Sea, similar studies have been conducted for the invasive crab species, namely the blue crab *Callinectes sapidus* [60,61,62] and the blue swimmer crab *Portunus segnis* (Forskål, 1775) (formerly known as *P. pelagicus*) [60]. 

The authors wish to discuss and encourage, based on the findings of the present study, the consumption of the non-indigenous *P. aztecus* over the vulnerable *P. elephas*. The findings of the present study clearly indicate that the *P. aztecus* fillets have higher content of protein, lipids, and energy in comparison to the *P. elephas* specimens. Moreover, the biggest *P. aztecus* fished individual was approximately 350 g. Therefore, the prawn’s tail fillet could be similar in size to the lobster’s tail fillet, covering several of the gastronomical demands of hotels and restaurants in which spiny lobsters tend to be sold [19].

It is well documented that coastal ecosystems are particularly vulnerable to anthropogenic actions [63,64], reducing marine biodiversity and contributing to overfishing [65,66], and the Mediterranean is no exception [67]. Unless we immediately implement corrective measures, it is most possible that many stocks of fish species of commercial interest will be virtually extinct by 2050, causing major disruptions in the global ecosystem.

## Figures and Tables

**Table 1:** Fillet proximate composition (percentage of wet weight) and statistical comparison (test statistic and associated *p*-value) of the European spiny lobster (*Palinurus elephas*) and northern brown shrimp (*Penaeus aztecus*). Data are means ±standard deviation (*n* = 10).

Proximate Composition	Spiny Lobster	Northern Brown Shrimp	Test Statistic and Associated Probability
Crude protein	19.31 ± 0.56	22.30 ± 0.27	5.20, *p* < 0.001
Crude fat	0.38 ± 0.06	0.51 ± 0.04	3.78, *p* < 0.001
Moisture	76.68 ± 2.56	74.39 ± 2.88	48.00, *p* < 0.001
Ash	2.13 ± 0.59	1.73 ± 0.07	−2.96, *p* < 0.05
Gross energy (kJ/g)	4.86 ± 0.13	5.52 ± 0.03	3.96, *p* < 0.05

**Table 2:** Proximate composition (percentage of wet weight), of decapod crustacean species in the Mediterranean, or imported live, and statistical comparison with values estimated for the European spiny lobster (*Palinurus elephas*). Data are mean values. Significance level (ns: non-significant, *: *p* < 0.05, **: *p* < 0.01, ***: *p* < 0.001).

Group	Species	Moisture	Ash	Protein	Lipid	Energy kJ/g	Reference
Lobsters	*Nephrops norvegicus*	74.95 **	2.15 ^ns^	20.80 ^ns^	0.15 ***	4.03 ***	[2]
	*Homarus gammarus*	78.65 **	1.90 ^ns^	17.95 ^ns^	0.40 *	3.56 ***	[3]
	*Homarus americanus*	79.85 ***	1.85 ^ns^	16.35 *	0.65 ***	3.36 ***	[3]
Prawns	*Aristeus antennatus*	74.05 ***	2.00 *	21.70 ^ns^	0.20 ***	4.21 ***	[1]
	*Parapenaeus longirostris*	74.20 ***	2.00 ^ns^	21.05 ^ns^	0.25 ***	4.12 ***	[1]
	*Penaeus aztecus*	76.81 ^ns^	1.69 *	19.23 ^ns^	0.94 ***		[41]
	*Penaeus kerathurus*	78.20 *	1.60 ^ns^	15.60 ***	2.10 ***		[4]
	*Penaeus kerathurus*	76.41 ^ns^	1.96 ^ns^	16.44 ***	1.64 ***	3.97 ***	[42]
	*Penaeus kerathurus*	76.61 ^ns^	1.59 *	20.21 ***	2.02 ***		[43]
	*Metapenaeus monoceros*	79.26 ***	1.68 ^ns^	18.04 ***	2.18 ***		[43]

**Table 3:** Proximate composition (percentage of wet weight) of decapod crustacean species in the Mediterranean, or imported live, and statistical comparison with values estimated for northern brown shrimp (*Penaeus aztecus*). Data are mean values. Significance level (ns: non-significant, *: *p* < 0.05, **: *p* < 0.01, ***: *p* < 0.001).

Group	Species	Moisture	Ash	Protein	Lipid	Energy kJ/g	Reference
Lobsters	*Nephrops norvegicus*	74.95 ^ns^	2.15 ***	20.80 ***	0.15 ***	4.03 ***	[2]
	*Homarus gammarus*	78.65 ***	1.90 ***	17.95 ***	0.40 *	3.56 ***	[3]
	*Homarus americanus*	79.85 ***	1.85 ***	16.35 ***	0.65 ***	3.36 ***	[3]
Prawns	*Aristeus antennatus*	74.05 ^ns^	2.00 ***	21.70 ***	0.20 ***	4.21 ***	[1]
	*Parapenaeus longirostris*	74.20 ^ns^	2.00 ***	21.05 ***	0.25 ***	4.12 ***	[1]
	*Penaeus aztecus*	76.81 **	1.69 ***	19.23 ***	0.94 ***		[41]
	*Penaeus kerathurus*	78.20 ***	1.60 ***	15.60 ***	2.10 ***		[4]
	*Penaeus kerathurus*	76.41 *	1.96 ***	16.44 ***	1.64 ***	3.97 ***	[42]
	*Penaeus kerathurus*	76.61 *	1.59 ^ns^	20.21 ***	2.02 ***		[43]
	*Metapenaeus monoceros*	79.26 ***	1.68 ***	18.04 ***	2.18 ***		[44]

**Table 4:** Fatty acid composition (percentage of total fatty acids) of the muscle tissue of *Palinurus elephas* and *Penaeus aztecus*.

Fatty Acid	*Palinurus elephas*	*Penaeus aztecus*
14:0	1.54 ± 0.26	1.20 ± 0.33
15:0	1.61 ± 0.22	1.48 ± 0.33
16:0	23.17 ± 3.80	22.77 ± 4.06
18:0	14.48 ± 3.43	16.30 ± 3.33
20:0	0.89 ± 0.26	1.12 ± 0.28
22:0	0.94 ± 0.19	0.93 ± 0.36
SFA	42.67 ± 7.67	43.98 ± 7.56
16:1n-7	9.14 ± 1.17 **	6.26 ± 1.50 **
18:1n-9	17.47 ± 4.23	13.55 ± 2.81
18:1n-7	4.25 ± 0.55	5.01 ± 1.45
20:1n-9/n-11	1.38 ± 0.46	1.58 ± 0.39
22:1n-9/n-11	0.37 ± 0.08	0.46 ± 0.17
24:1n-9	0.24 ± 0.06	0.52 ± 0.24
MUFA	32.30 ± 6.16	28.13 ± 4.48
18:2n-6	2.66 ± 0.53	2.49 ± 0.83
20:2n-6	1.80 ± 0.39 *	0.97 ± 0.62 *
20:3n-6	0.17 ± 0.05	n.d.
20:4n-6	17.85 ± 2.73 ***	7.66 ± 1.25 ***
22:4n-6	0.46 ± 0.12	0.65 ± 0.31
22:5n-6	1.08 ± 0.32	1.08 ± 0.31
Total n-6	23.14 ± 3.13 ***	12.05 ± 1.31 ***
18:3n-3	0.78 ± 0.13	0.81 ± 0.11
18:4n-3	0.18 ± 0.04	0.55 ± 0.28
20:3n-3	0.22 ± 0.05	0.24 ± 0.05
20:4n-3	0.17 ± 0.02	0.18 ± 0.03
20:5n-3	1.23 ± 0.38 **	9.05 ± 3.06 **
22:5n-3	0.57 ± 0.31	1.14 ± 0.68
22:6n-3	0.95 ± 0.40 **	7.62 ± 1.34 **
Total n-3	2.24 ± 0.88 **	18.18 ± 3.03 **
n-3/n-6	0.10 ± 0.03 ***	1.18 ± 0.58 ***

Note: SFA, total saturated fatty acids; MUFA, total monounsaturated fatty acids; n.d., not detected. *: *p* < 0.05, **: *p* < 0.01, ***: *p* < 0.001.
